# Role of Subcutaneous Adrenaline in Reducing Bleeding Risks During Chest Drain Insertion in High-Risk Patients: A Case Report

**DOI:** 10.7759/cureus.91078

**Published:** 2025-08-26

**Authors:** Joshua Martin Manogaran, Mamoun Ibrahim

**Affiliations:** 1 General Internal Medicine, Hillingdon Hospital, London, GBR; 2 Respiratory Medicine, Hillingdon Hospital, London, GBR

**Keywords:** bleeding risk, cavitary pneumonia, chest drain, emergency chest drain, pleural disease, pleural interventions, pneumonia, rare cause of pleural effusion

## Abstract

The vasoconstrictive effect of adrenaline combined with lidocaine is well-documented, but its efficacy in reducing procedure-related bleeding during chest drain insertion in coagulopathic patients remains unreported. We present the case of a 37-year-old male with alcohol-related decompensated liver cirrhosis (Child-Pugh C), thrombocytopenia (23,000 × 10⁹/L), and elevated INR (2.3), who developed a spontaneous right hydropneumothorax secondary to cavitary pneumonia. Despite profound coagulopathy, a lifesaving 18F Seldinger chest drain was inserted using adrenaline-lidocaine (1:200,000) for local anesthesia, achieving immediate hemostasis with no periprocedural bleeding. This highlights the potential role of adrenaline in mitigating bleeding risks during invasive procedures in high-risk patients.

## Introduction

Decompensated liver disease poses a significant risk of bleeding, especially during invasive procedures. This happens due to an apparent reduction in coagulation factors, including II, V, VII, IX, X, and XI, which are derived from the liver [1]. Furthermore, this is accompanied by a decreased platelet count and prolonged coagulation screening, particularly as indicated by the international normalized ratio (INR)/prothrombin time (PT) [1]. While prophylactic blood product administration (e.g., fresh frozen plasma (FFP), platelets) is standard, emergent scenarios may preclude optimization. Adrenaline’s α-adrenergic vasoconstrictive effects, combined with lidocaine’s anesthetic properties, may reduce bleeding risks, but evidence in pleural procedures is lacking.

We report a case of a 37-year-old gentleman with decompensated liver cirrhosis and significantly impaired coagulation, with a platelet count of 23 × 10⁹/L and an INR of 2.3, secondary to end-stage alcoholic liver cirrhosis. Despite impaired coagulation, clinical worsening necessitated an urgent, life-saving chest drain insertion. We aim to discuss the challenges faced by clinicians in these critical scenarios and provide evidence to suggest an overall improvement in patient outcomes.

## Case presentation

**Figure 1:**
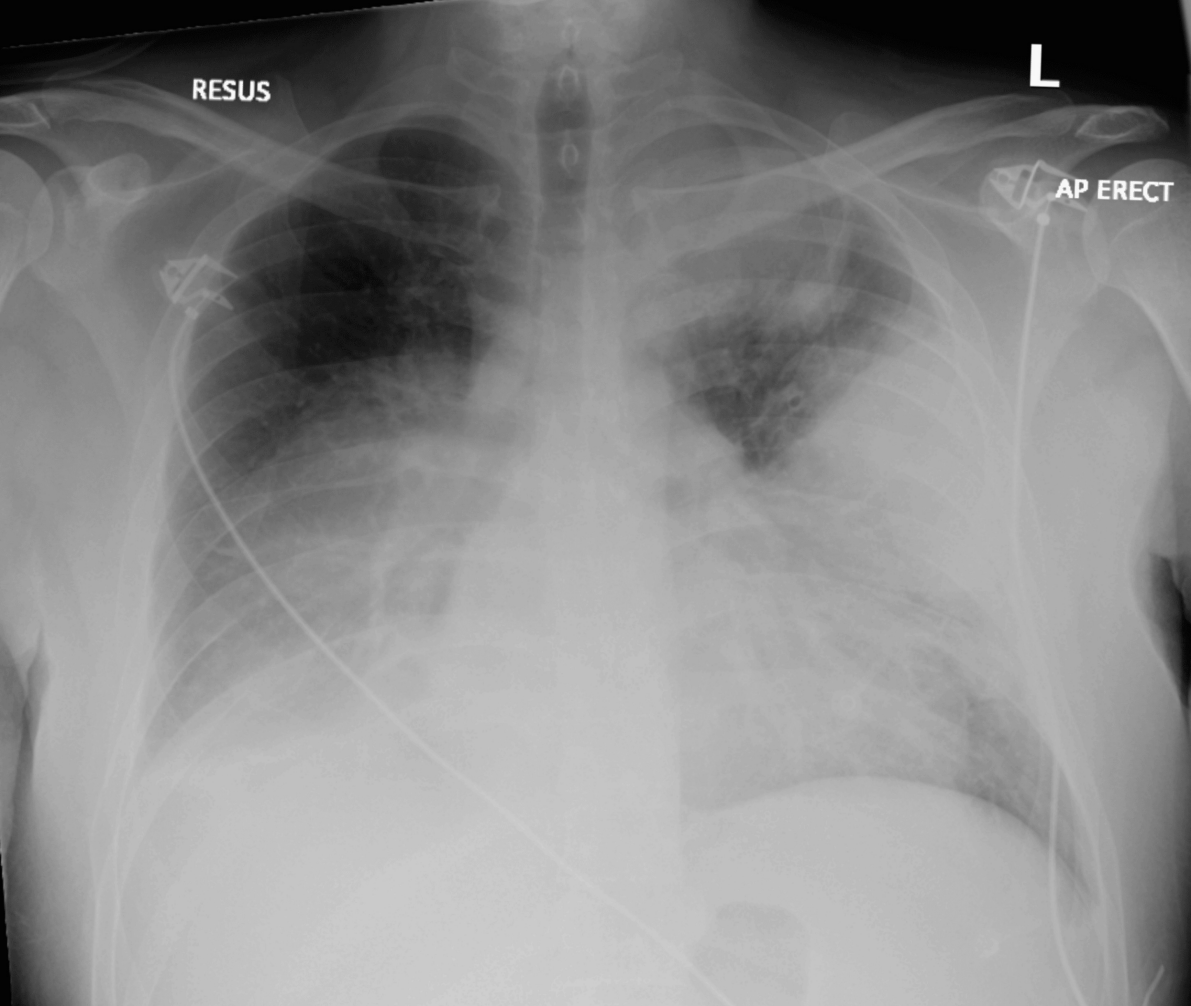
Initial chest X-ray in the ED Highly abnormal appearances of the lungs with dense, consolidative masses bilaterally. Lymphoma is included in the differential diagnosis, although the appearances are nonspecific. ED: emergency department

A 37-year-old male presented to the emergency department (ED) of a secondary care hospital with a four-day history of high-grade intermittent fever associated with productive cough and greenish sputum. His past medical history includes alcohol-related liver cirrhosis, for which he was on the transplant list. On examination, the patient was tachypneic, with increased work of breathing and widespread coarse crackles over the chest. The initial chest X-ray (Figure 1) in the ED showed bilateral dense opacities on the left more than the right. Following resuscitation, empirical intravenous co-amoxiclav was initiated, and a blood culture was sent, which yielded *Streptococcus pneumoniae* within 24 hours, along with a positive pneumococcal antigen test in the urine.

**Figure 2:**
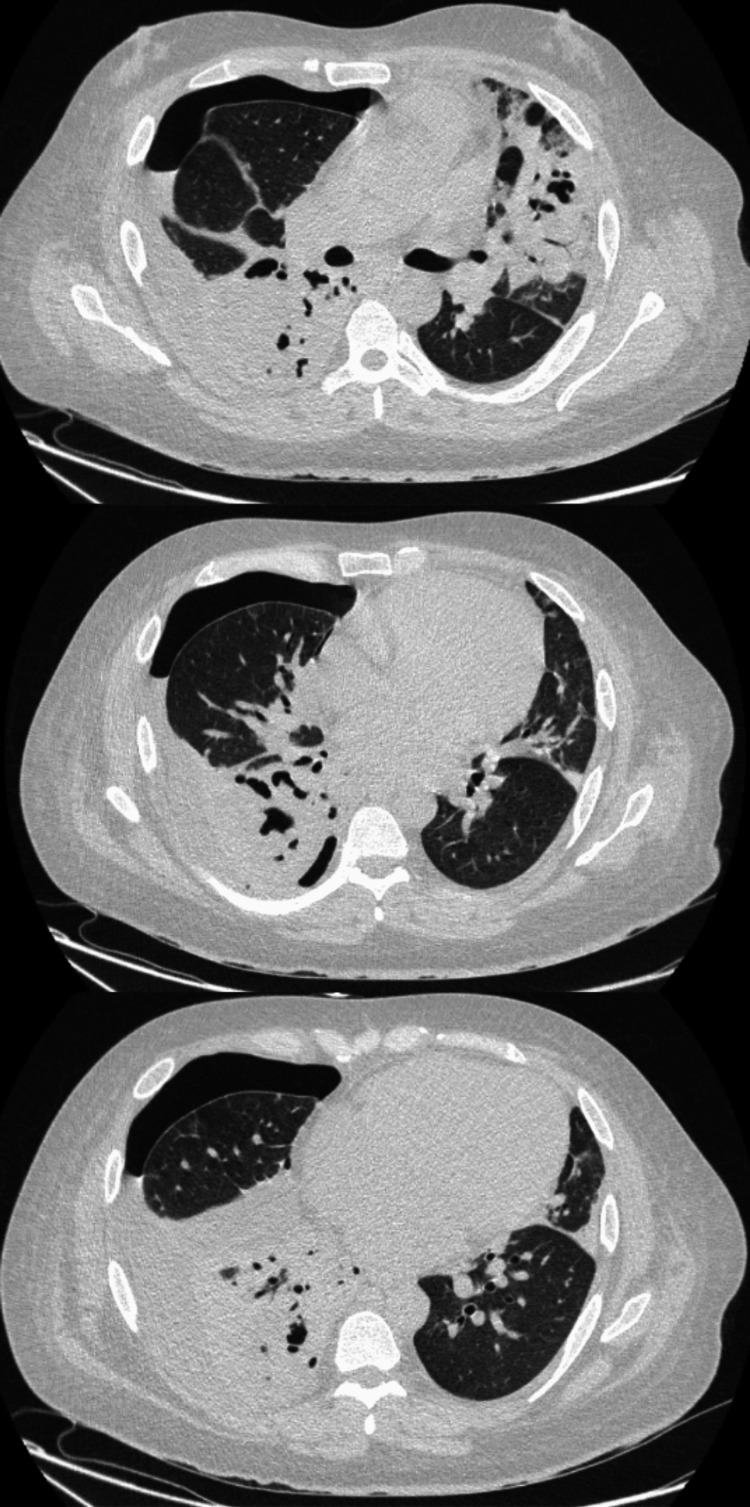
CT scan of the thorax Bilateral areas of mass-like consolidation predominantly on the right, accompanied by extensive cavitation. A new right-sided hydropneumothorax has developed, presumably secondary to pleural fistulation. Overall, the appearances are most likely to represent extensive cavitating pneumonia. CT: computed tomography

On day 2 of admission, the patient was transferred to the intensive care unit (ICU) with a diagnosis of worsening sepsis secondary to pneumonia, as evidenced by worsening type 1 respiratory failure, and co-amoxiclav was continued based on the blood culture sensitivities. During his stay in the ICU, a CT thorax (Figure 2) was performed on day 5 of admission to characterize further the organized, mass-like opacity observed on initial chest radiography.

A multidisciplinary team (MDT) discussion involving radiology and respiratory specialists concluded that the hydropneumothorax was most likely secondary to a parenchymal-pleural fistula. He continued to improve gradually while remaining under the care of the ICU.

**Figure 3:**
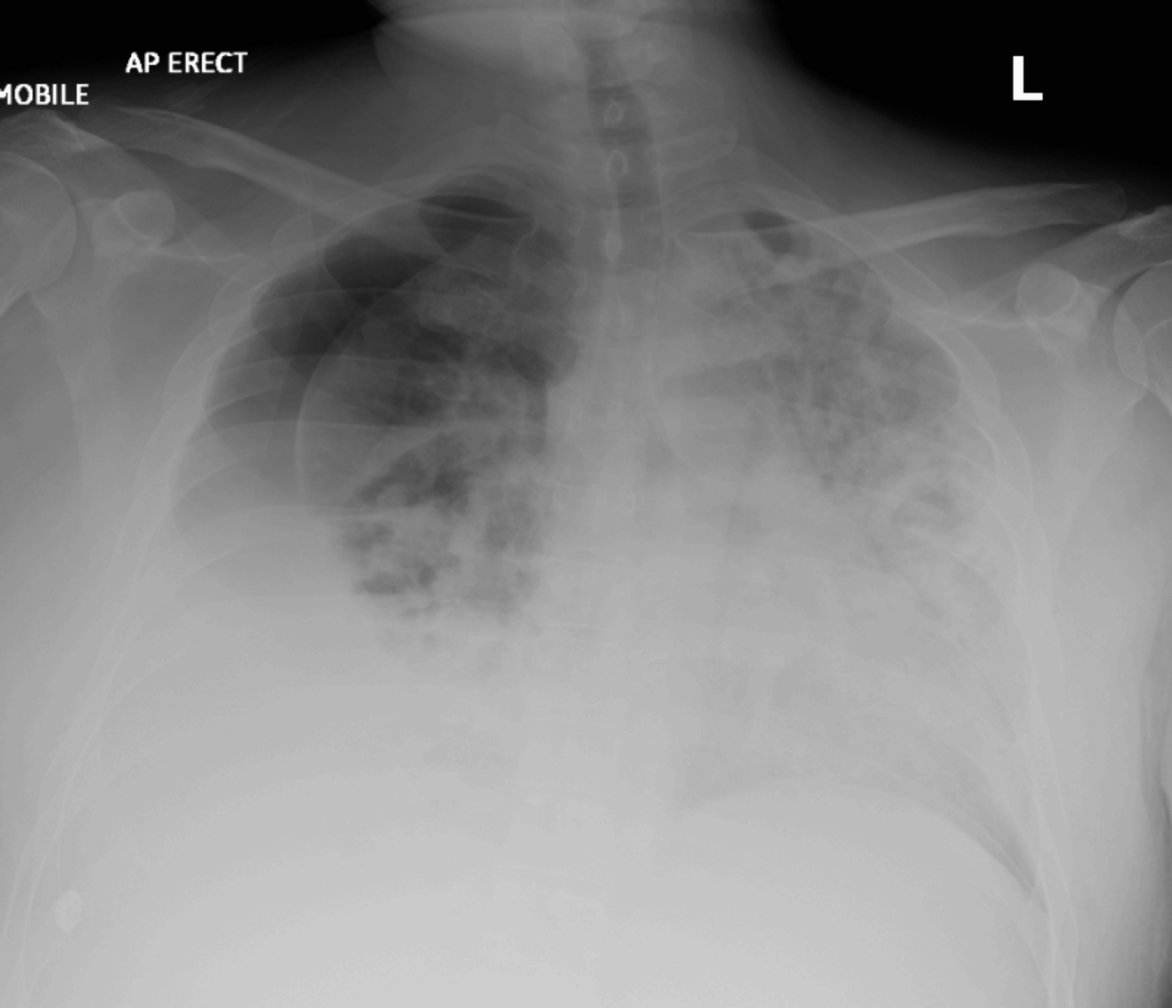
Chest X-ray Large right-sided pneumothorax on a background of consolidation throughout the remaining visible lung field. Overall appearance is consistent with features of rapidly progressing pneumonia with a large coexisting pneumothorax.

Subsequently, the patient was stepped down from the ICU to the respiratory ward on day 6 of admission. However, on day 8, he experienced sudden clinical deterioration, presenting with fever, increased work of breathing, confusion, and worsening type 1 respiratory failure. An urgent chest radiograph (Figure 3) demonstrated progression of the hydropneumothorax. Additionally, an isolated episode of melena was noted on the ward.

Laboratory investigations (Table 1) revealed a significant coagulopathy, characterized by platelet counts of 23 × 10⁹/L, an INR of 2.3, and a hemoglobin level of 67 g/L. Following clinical assessment and MDT discussion with the hematology team, an emergency decision was made to insert a chest drain due to worsening hydropneumothorax. Despite the high risk of bleeding, the procedure was deemed life-saving. The patient’s family was informed, and after obtaining consent, a chest drain was inserted with all appropriate precautions.

**Table 1:** Blood investigation showing deranged coagulation, anemia, neutrophilia, and thrombocytopenia INR: international normalized ratio, WBC: white blood cell, APTT: activated partial thromboplastin time

Blood results	Value	Reference range
Hemoglobin	67 g/L	130-168 g/L
WBC	16.7 x 10⁹/L	4.2-10.6 x 10⁹/L
Neurophils	14.8 x 10⁹/L	2-7.1 x 10⁹/L
Lymphocytes	0.3 x 10⁹/L	1.1 x 3.6 x 10⁹/L
Platelets	23 x 10⁹/L	130-370 x 10⁹/L
INR	2.3	-
APTT	34 seconds	12.8-17.4 seconds

Chest drain insertion

Following hematology advice, the patient was transfused with one pool of platelets and FFP at 10-15 mL/kg approximately one hour before the procedure to reduce the risk of peri- and post-procedural bleeding.

Thoracic ultrasound was used to identify a safe insertion site, with the patient positioned in the left lateral decubitus position. After sterile skin preparation, approximately 35 mL of 1% lidocaine with adrenaline (1:200,000) was infiltrated into the skin, subcutaneous tissue, and deeper layers to ensure adequate anesthesia and hemostasis. The patient's weight was around 100 kg.

An introducer needle and guide wire were then inserted without resistance. A 1-1.5 cm skin incision was made, followed by serial insertion of three dilatators. No bleeding was observed throughout the process. Using the Seldinger technique, an 18F chest drain was successfully inserted and secured to the skin. The patient experienced immediate symptomatic relief. Hemostasis was achieved, and no peri-procedural complications occurred.

Approximately 1 liter of brown, turbid pleural fluid was drained immediately. Analysis revealed (Table 2) a pleural fluid lactate dehydrogenase (LDH) of 6,439 U/L (serum LDH: 343 U/L), pleural fluid protein of 38 g/L, and serum protein of 58 g/L. Cytological examination revealed a cellular sample predominantly composed of neutrophils, with a mixed population of lymphocytes and macrophages. The findings were consistent with acute inflammation and suggestive of empyema, with no malignant cells identified. Pleural fluid cultures showed no microbial growth. The patient remained clinically stable over the following three days.

**Table 2:** Pleural fluid analysis LDH: lactate dehydrogenase

Test	Value	Reference range
Gram stain	White blood cells +++	-
Differential	80% polymorphs, 20% mononuclear cells	-
Culture	No organism	-
Pleural fluid LDH	6,439 U/L	-
Pleural fluid protein	38 g/L	-
Serum LDH	343 U/L	125-243 U/L
Serum protein	58 g/L	60-80 g/L

Further care

Subsequently, the patient’s condition further deteriorated, requiring ICU input. On day 9, he developed acute type 2 respiratory failure and was intubated and ventilated. He was transferred to a tertiary center under the joint care of the ICU and hepatology teams for specialist input and management. Intravenous meropenem was commenced on day 18 of admission. The patient showed gradual clinical improvement, with resolution of the infection, and was extubated without complications on day 21.

He was repatriated to our hospital on day 43 and readmitted to the respiratory ward. The infectious diseases team recommended continuing intravenous meropenem for a total of six weeks. The patient ultimately survived the acute episode and was discharged on day 55 under the care of the outpatient parenteral antimicrobial therapy team to complete the antibiotic course.

**Figure 4:**
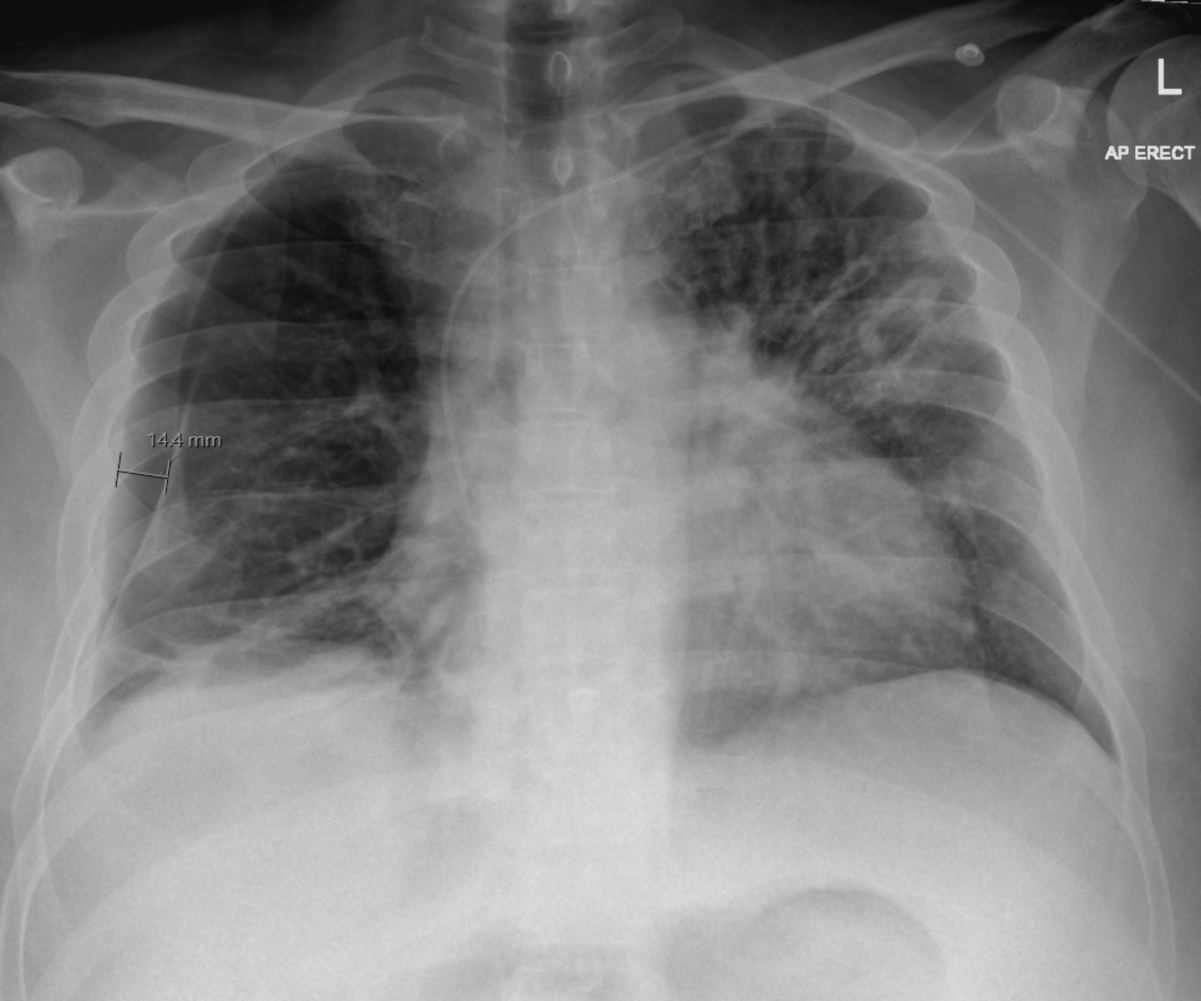
Chest X-ray taken a few days before discharge

During his admission, he received regular input from the palliative care team due to a guarded prognosis. He also experienced significant deconditioning from prolonged immobility. Upon discharge, follow-up care with the community palliative care team was arranged through the district nurse. In agreement with the hepatology team, it was decided that respiratory follow-up would be pursued only if there was a substantial improvement in his health condition. At the time of writing, the patient remained under evaluation and on the list for liver transplantation. A chest x-ray taken a few weeks prior to discharge (Figure 4) shows improvement in the pneumothorax.

**Figure 5:**
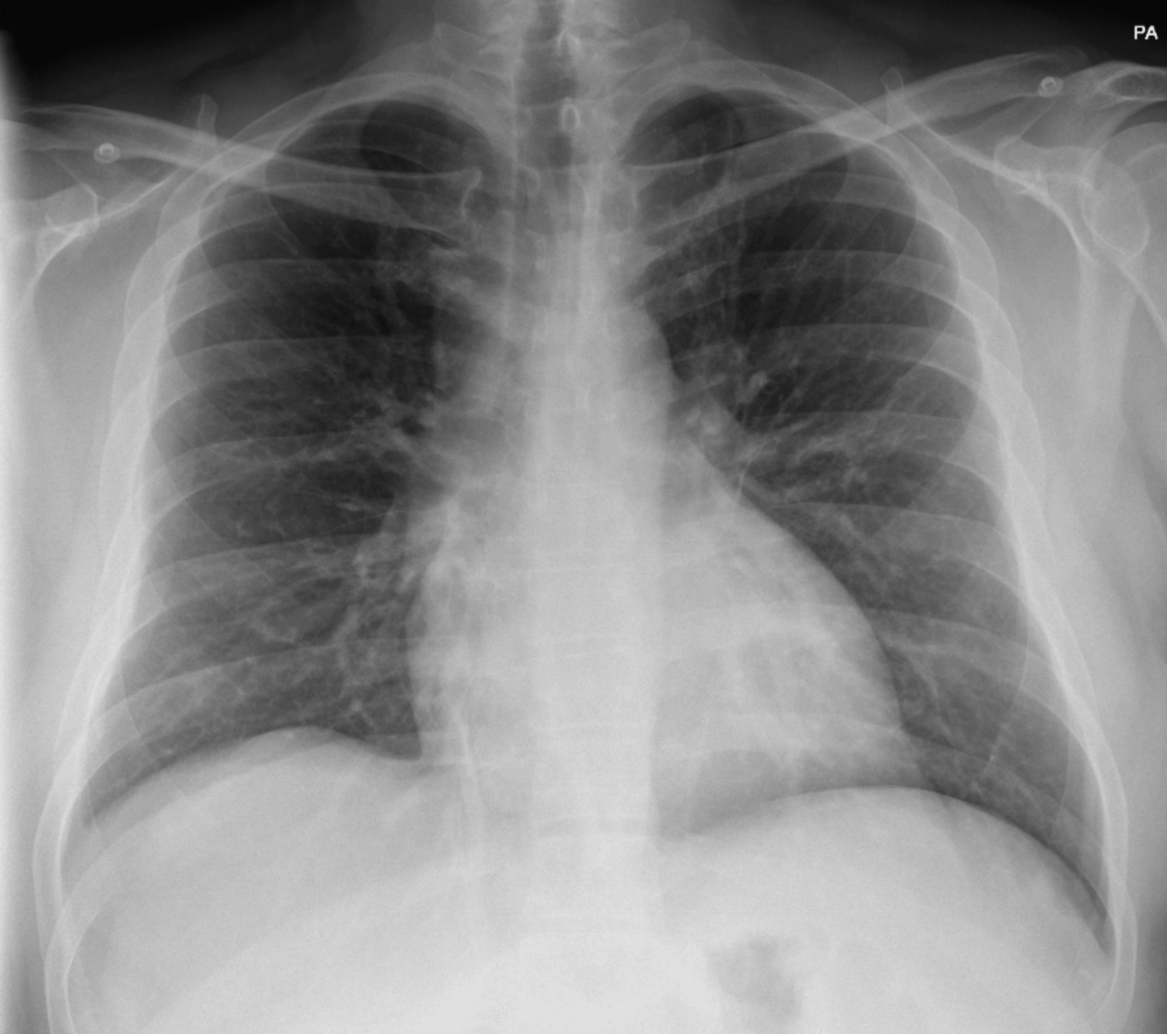
Chest X-ray showing clear lung fields with complete resolution

A follow-up chest X-ray (Figure 5), performed nearly a year later during a subsequent admission for presumed community-acquired pneumonia, showed complete resolution of the previous bilateral pneumonia and hydropneumothorax.

## Discussion

Lidocaine combined with adrenaline has long been used in clinical practice. The addition of adrenaline significantly prolongs the duration of local anesthesia by inducing vasoconstriction through activation of alpha-adrenergic receptors in the vascular smooth muscle [2]. This vasoconstrictive effect also serves to minimize bleeding during procedures. The use of lidocaine with adrenaline at a concentration of 1:200,000 permits higher doses, up to 7 mg/kg (maximum 500 mg or 50 mL of 1% lidocaine), to be safely administered [3]. This was used instead of the usual recommendation of lidocaine infiltration around the periosteum [4].

In our patient, hemostasis is profoundly impaired secondary to coagulopathy, with an elevated INR (e.g., 2.3) and severe thrombocytopenia (e.g., platelet count 23 × 10⁹/L), secondary to decompensated chronic liver disease. This increases the risk of uncontrolled bleeding during invasive procedures such as chest drain insertion. Ultrasound was used to mark the safe site of insertion for chest drain insertion, as this can potentially prevent significant harm due to improper site selection and decrease procedure-related risks [5,6].

Current guidelines of the British Society of Haematology (BSH) [2] indicate that elective chest‑drain insertion in patients with coagulopathy (INR >1.5 or platelets <50 × 10⁹/L) should be deferred until laboratory parameters are corrected. Nonetheless, in emergencies or for low‑risk procedures under ultrasound guidance, intervention may proceed safely without full hemostatic correction, provided a multidisciplinary discussion and informed consent are documented.

For moderate-risk procedures, such as chest drain insertion, BSH guidance recommends correcting platelet counts to ≥50 × 10^9/L and INR <1.5 [2]. In this patient (platelets 23 × 10⁹/L; INR 2.3), these criteria were not met. Nonetheless, given the life-threatening nature of his medical condition secondary to worsening respiratory failure, an urgent chest drain insertion was opted for. When such patients require urgent intervention, there may be insufficient time to correct coagulation parameters with blood products. Hence, a chest drain was inserted emergently following MDT approval, with complete discussion of bleeding risk and administration of platelets and FFP. This approach aligns with guideline-permitted flexibility for urgent interventions. There was minimal to no bleeding during the procedure while cutting through the subcutaneous and deeper tissues, highlighting the role of lidocaine with adrenaline as the infiltration drug of choice.

This case highlights that, in these high-risk scenarios, the use of an adrenaline-lidocaine mixture, as opposed to lidocaine alone, may reduce the likelihood of life-threatening hemorrhage during the procedure. This report is based on a single-case observation; therefore, controlled studies are needed to provide further evidence and clarity. Additionally, the confounding effects of pre-procedure administration of FFP and/or platelets cannot be ruled out.

## Conclusions

In coagulopathic patients requiring emergency pleural procedures, the use of an adrenaline-lidocaine mixture may be considered to minimize bleeding. In life-threatening scenarios, procedural risks may outweigh concerns about bleeding; however, adjuncts such as adrenaline can help mitigate these hazards. Optimal outcomes in complex cases require the involvement of an MDT, including radiologists, hematologists, and respiratory physicians. Early cardiothoracic input is also essential; if this service is not available, transfer to a tertiary care center should be considered to address complications promptly. Finally, obtaining informed consent with the active involvement of the patient and their next of kin is essential, particularly in high-risk, life-threatening emergencies. Senior or experienced consultants should be directly involved in performing complicated procedures.
